# Identification of optimal endogenous reference RNAs for RT-qPCR normalization in hindgut of rat models with anorectal malformations

**DOI:** 10.7717/peerj.6829

**Published:** 2019-04-23

**Authors:** Caiyun Long, Yunxia Xiao, Siying Li, Xiaobing Tang, Zhengwei Yuan, Yuzuo Bai

**Affiliations:** 1 Department of Pediatric Surgery, Shengjing Hospital of China Medical University, Shenyang, Liaoning Province, China; 2 The Key Laboratory of Health Ministry for Congenital Malformation, Shengjing Hospital of China Medical University, Shenyang, Liaoning Province, China

**Keywords:** Anorectal malformations, Reference gene, Stability, Variability, PCR

## Abstract

**Background:**

Quantitative real-time polymerase chain reaction (RT-qPCR) is a sensitive method for quantifying mRNA abundance. With relative expression analysis, however, reliable data output is dependent on stably expressed reference genes across the samples being studied. In anorectal malformations (ARMs), there is limited data on the selection of appropriate reference genes.

**Purpose:**

This study was aimed to investigate the optimal reference genes for PCR in ARM rat models.

**Methods:**

We selected 15 commonly used reference genes (Rps18, Actb, B2m, Gapdh, Ppia, Hprt1, Pgk1, Ywhaz, Tbp, Ubc, Rps16, Rpl13a, Rplp1, Sdha, and Hmbs) as candidate reference genes and detected their mRNA expression in ARM samples by RT-qPCR. The expression stability and variability of these transcripts were subsequently evaluated using four methods (geNorm, NormFinder, comparative ΔCt, and BestKeeper).

**Results:**

The abundance of the candidate reference genes was qualified by RT-qPCR and the cycle threshold (Ct) values ranged between 14.07 (Rplp1) and 21.89 (Sdha). In the overall candidate genes, different variations existed across the different algorithms. A comprehensive analysis revealed that Rpl13a ranked first among the relatively stable genes, followed by Ywhaz, Rps18, Sdha, and Hmbs.

**Conclusions:**

The most stable reference genes for RT-qPCR were Rpl13a, Ywhaz, and Rps18 in ETU-induced ARMs in rat fetus. This study provided a foundation for reference gene selection for future gene expression analyses.

## Introduction

Anorectal malformations (ARMs) are common congenital gastrointestinal malformations manifested by anal stenosis, ectopic anus, and urethrorectal fistula (Bai et al., 2004; De Blaauw et al., 2013; Wijers et al., 2014). The specific mechanism of ARMs still remains unclear although numerous studies have revealed that various genes are involved in the development of this disease (Draaken et al., 2012; Wijers et al., 2014). As clinical samples are difficult to obtain, animal models are commonly used to study ARMs. The most commonly used model is ETU-induced ARMs in rat fetus (Macedo, Martins & Meyer, 2007; Qi, Beasley & Frizelle, 2002).

In ARM-related studies, it is inevitable to analyze gene expression. Quantitative real-time polymerase chain reaction (RT-qPCR) is commonly used because of its high efficiency and sensitivity. While analyzing target gene expression by RT-qPCR, there are many strategies by which relative quantification is often adopted (Yuan et al., 2006). In this course, normalization to extensively and stably expressed endogenous reference genes is commonly used to reduce the variations caused by input RNA amount or reverse transcription efficiency (Bustin et al., 2009; Giulietti et al., 2001).

It is noteworthy that this approach is based on the presupposition that the transcript abundance of selected reference genes is stable in all samples under various conditions (Giulietti et al., 2001). However, researchers have found that what was previously thought as “stably expressed” reference genes are not actually strictly invariant. In fact, the reference genes can be affected by various factors (Harris, Reeves & Phillips, 2009; Mughal et al., 2018). For instance, diseases or experimental treatments can alter the expression stability of the reference genes (Aggarwal et al., 2018; Giulietti et al., 2001; Neerukonda et al., 2016). Physical or chemical factors can also influence the variability of the reference genes (Giulietti et al., 2001; Mahanty et al., 2017; Svingen et al., 2015; Yuanyuan et al., 2015). Moreover, the development stage or even seasonal factor has an effect on the optimal reference gene selection (Liu et al., 2014; Macabelli et al., 2014; Mughal et al., 2018). The reference gene expression instability may be caused by organ composition and cell types (Guenin et al., 2009).

Based on the above conditions, some researchers have suggested that normalization against a reference gene should not be acceptable unless it has been clearly confirmed that the reference gene is invariantly expressed in the experimental and control conditions (Aggarwal et al., 2018; Bustin et al., 2009). In order to obtain reliable results in RT-qPCR, systematic validation of reference genes is essential (Guenin et al., 2009).

To the best of our knowledge, there is no study identifying the optimal reference genes in ETU-induced ARM rats. Therefore, systematic evaluation is essential for obtaining accurate RT-qPCR results. In this study, we analyzed the expression of 15 candidate reference genes in the hindgut of normal and ETU-induced ARMs in rat fetuses during critical time points for anorectal development (Bai et al., 2004; Faria, De Jesus Simoes & Martins, 2015; Macedo, Martins & Meyer, 2007). In addition, we systematically evaluated and ranked the expression stability of these genes aiming to identify an optimal reference gene for RT-qPCR in ARM rat models.

## Materials and Methods

### Ethical statement

This study was approved by the China Medical University Ethics Committee (no. 2015 PS213K). All procedures were performed according to the guidelines for the care and use of laboratory animals.

### Animal models and sample preparation

A total of 18 mature female Wistar rats (age: 7–9 weeks old, body weight: 250–300 g) were provided by the Experimental Animal Center of the Shengjing Hospital of China Medical University (Shenyang, Liaoning, China). The animals were raised in a specific pathogen-free environment of The Key Laboratory of Health Ministry for Congenital Malformations (Shenyang, Liaoning, China). Surgeries were performed after euthanasia by intraperitoneally injecting sodium pentobarbital, and all efforts were made to minimize the pain and suffering of the animals.

Anorectal malformations models were developed according to an earlier report (Bai et al., 2004; Long et al., 2018). Briefly, nine pregnant rats were administered 125 mg/kg of 1% ETU (Sigma-Aldrich, Merck Millipore, Darmstadt, Germany) by single gavage on GD10 (gestational day (GD); GD0, presence of sperm in vaginal smear after overnight mating), while the other nine rats received normal saline as control. The presence of ARMs was determined by microscopy. The hindguts of the embryos were removed from the surrounding tissues (Fig. S1). The tissues were then immediately frozen in liquid nitrogen for RT-qPCR.

A total of 123 ETU-induced ARM fetuses (A) and 115 saline-treated normal fetuses (N) were obtained by cesarean section on GD16. The incidence of ARMs in the ETU-treated fetuses was 85.8% (97/113). All hindgut samples from each age group were immediately frozen in liquid nitrogen for subsequent RNA extraction.

### RNA extraction and cDNA synthesis

The total RNA was isolated using the miRNeasy Mini kit (Qiagen GmbH, Hilden, Germany) according to the manufacturer’s instructions. RNA was qualified and quantified using Tecan Infinite 200 multimode plate reader (Tecan AG, Hombrechtikon, Switzerland) and formaldehyde agarose gel electrophoresis. Only samples with RNA absorption ratios of A260/280 ranging from 1.9 to 2.1 and RNA integrity number ≥7 from the Agilent 2200 RNA assay (Agilent Technologies, Inc., Santa Clara, CA, USA) were used for cDNA synthesis. The cDNA was synthesized from 500 ng of total RNA in a final volume of 10 μl using PrimeScript RT reagent kit (Takara Biotechnology Co. Ltd., Dalian, China) according to the manufacturer’s instructions.

### Selection of the candidate reference genes

A total of 15 commonly used candidate genes (Rps18, Actb, B2m, Gapdh, Ppia, Hprt1, Pgk1, Ywhaz, Tbp, Ubc, Rps16, Rpl13a, Rplp1, Sdha, and Hmbs) were assessed to identify the most stably expressed and optimal reference genes in studies of ARMs. All the primers were designed and synthesized by Sangon (Sangon Biotech Co. Ltd., Shanghai, China).

### Amplification by RT-qPCR

The PCR efficiency of each primer pair was tested using calibration curves, and the efficiency rate was calculated according to the following equation: PCR amplification efficiency = 10^−1/slope^ − 1 (Bustin et al., 2009). The gene expression levels were assessed using SYBR Premix Ex Taq II kit (Takara Biotechnology Co. Ltd., Dalian, China). Each reaction system contained 2 μl cDNA template, 0.8 μl of each forward and reverse primers (10 nM), 6 μl RNase-free water, 0.4 μl ROX II, and 10 μl SYBR Green premix Ex Taq II (Tli RNaseH Plus) in a final volume of 20 μl. The amplification cycles were set as follows: denaturation at 95 °C for 30 s followed by 40 cycles of 95 °C for 5 s and 60 °C for 34 s on an ABI 7500 detection system (Applied Biosystems, Carlsbad, CA, USA). Nine biological replications were used for each group and three technical replications for each biological replicate (Table S1).

### Assessment of gene expression

To reduce bias resulting from a single method, four software including geNorm, NormFinder, comparative ΔCt, and BestKeeper were used to analyze the expression stability of the candidate genes. For input data of geNorm and NormFinder, the raw cycle threshold (Ct) values were transformed to quantify using 2^−ΔCt^ (ΔCt, Ct value of each gene minus the lowest Ct value of the corresponding gene in different samples). The geNorm applet calculates the gene expression stability value (*M* value) of the reference genes during stepwise exclusion of the least stable reference gene (Vandesompele et al., 2002). The genes were ranked by increasing expression stability, ending with the two most stably expressed genes. Next, the optimal number of genes was identified according to a pairwise variation value (*V* value) between the candidate genes. The optimal number of reference genes was considered acceptable when the *V* value was <0.15. NormFinder calculates the stability value of each gene as well as a combination of two genes to analyze the expression stability of the candidate genes (Andersen, Jensen & Orntoft, 2004). The corresponding estimated intragroup and intergroup variations are also provided. Using the ΔCt method, we calculated the ΔCt values for the pairwise genes and assessed the expression stability using the standard deviations (SD) of ΔCt values for each reference gene (Pfaffl et al., 2004). BestKeeper calculates the expression stability based on coefficient variance (CV) and SD based on the untransformed Ct values. BestKeeper also ranks the candidate genes according to the stability (Silver et al., 2006).

### Statistical analyses

All statistical analyses were performed using SPSS 21.0 software (IBM Corporation, Armonk, NY, USA). The data are presented as the mean ± SD unless indicated otherwise. All experiments were performed in at least three replicates.

## Results

### Selection of the candidate reference genes

In this study, 15 commonly used candidate RNA reference genes (He et al., 2013; Kim et al., 2011; Klenke et al., 2016; Ruedrich et al., 2013; Taki, Abdel-Rahman & Zhang, 2014) were investigated in the hindgut of both normal and ETU-induced ARMS in rat fetuses. Detailed information including full names, NCBI accession numbers, and position and PCR product lengths of these candidate genes are listed in Table 1. The sequences of forward and reverse primers used for RT-qPCR are listed in Table 2.

**Table 1:** Detailed information of 15 candidate reference genes evaluated in this study.

Genes	Full name	Accession No.	Position	Length (bp)	Amplification efficiency
Rps18	18S Ribosomal RNA	NM_213557	exon2/exon3	140	1.02
Actb	ß-actin	NM_031144	exon3/exon4	165	1.05
B2m	ß-2-microglobuIin	NM_012512	exon2/exon3, 4	114	1.06
Gapdh	Glyceraldehyde-3-phosphate dehydrogenase	NM_017008	exon6/exon7	92	0.99
Ppia	Peptidylprolyl isomerase A	NM_017101	exon1/exon3	133	1.04
Hprt1	Hypoxanthine phosphoribosyl transferase 1	NM_012583	exon8/exon9	105	1.02
Pgk1	Phosphoglycerate kinase 1	NM_053291	exon8/exon9	104	1.03
Ywhaz	Tyrosine 3-monooxygenase	NM_013011	exon2/exon3	127	1.00
Tbp	TATA binding protein	NM_001004198	exon4/exon5	123	1.01
Ubc	Ubiquitin C	NM_017314	exon3	82	0.99
Rps16	16S Ribosomal RNA	NM_001169146.1	exon1/exon3	147	1.05
Rpl13a	Ribosomal protein L13A	NM_173340	exon5/exon7	131	1.01
Rplp1	60S acidic ribosomal protein large PI	NM_001007604	exon2/exon3	87	0.98
Sdha	Succinate dehydrogenase complex, subunit A, flavoprotein (Fp)	NM_130428	exon9/exon10	105	0.99
Hmbs	Hydroxymethylbilane synthase	NM_013168	exon13/exon14	76	1.04

**Note:**

Accession No., NCBI accession number. PCR amplification efficiency = 10^−1/slope^ − 1.

**Table 2:** Primer sequences of 15 candidate reference genes.

Genes	Forward primer sequence (5′-3′)	Reverse primer sequence (5′-3′)
Rps18	AAGTTTCAGCACATCCTGCGAGTA	TTGGTGAGGTCAATGTCTGCTTTC
Actb	TGTCACCAACTGGGACGATA	GGGGTGTTGAAGGTCTCAAA
B2m	CGAGACCGATGTATATGCTTGC	GTCCAGATGATTCAGAGCTCCA
Gapdh	GACATGCCGCCTGGAGAAAC	AGCCCAGGATGCCCTTTAGT
Ppia	GTCAACCCCACCGTGTTCTTC	ATCCTTTCTCCCCAGTGCTCAG
Hprt1	TTGTTGGATATGCCCTTGACT	CCGCTGTCTTTTAGGCTTTG
Pgk1	ATGCAAAGACTGGCCAAGCTAC	AGCCACAGCCTCAGCATATTTC
Ywhaz	AGACGGAAGGTGCTGAGAAA	GAAGCATTGGGGATCAAGAA
Tbp	ACCGTGAATCTTGGCTGTAAAC	CGCAGTTGTTCGTGGCTCTC
Ubc	TCGTACCTTTCTCACCACAGTATCTAG	GAAAACTAAGACACCTCCCCATCA
Rps16	AAGTCTTCGGACGCAAGAAA	TGCCCAGAAGCAGAACAG
Rpl13a	GGATCCCTCCACCCTATGACA	CTGGTACTTCCACCCGACCTC
Rplp1	AAAGCAGCTGGTGTCAATGTT	GCAGATGAGGCTTCCAATGT
Sdha	TCCTTCCCACTGTGCATTACAA	CGTACAGACCAGGCACAATCTG
Hmbs	TCTAGATGGCTCAGATAGCATGCA	TGGACCATCTTCTTGCTGAACA

### Expression profiles of the candidate reference genes

**Figure 1:**
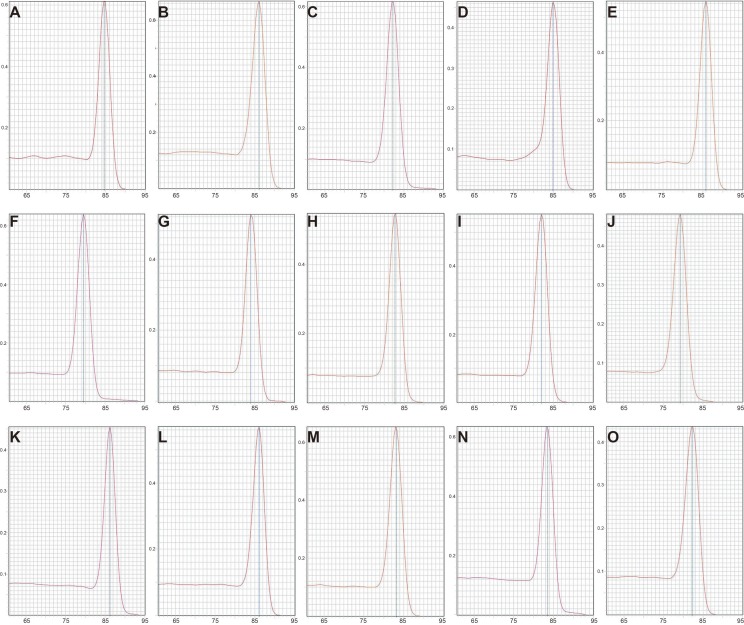
Melting curves of 15 candidate reference genes in RT-qPCR. (A) Rps18, (B) Actb, (C) B2m, (D) Gapdh, (E) Ppia, (F) Hprt1, (G) Pgk1, (H) Ywhaz, (I) Tbp, (J) Ubc, (K) Rps16, (L) Rpl13a, (M) Rplp1, (N) Sdha, (O) Hmbs. The *x* axis represents temperature and the *y* axis represents derivative reporter derived from ABI 7500 detection system. RT-qPCR was performed for nine biological replications in each group.

**Figure 2:**
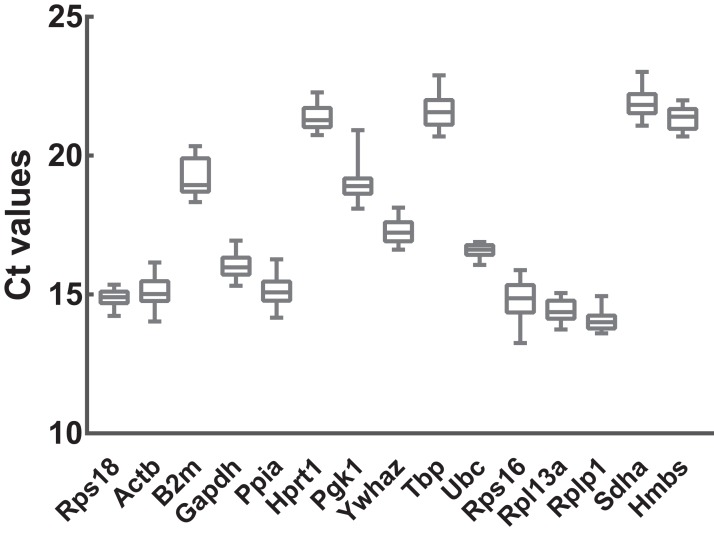
Transcript abundances of 15 candidate reference genes. Box and whiskers diagram shows the minimum, first quartile, median, third quartile, and maximum of Ct values from the bottom up. A total of 15 candidate reference genes are listed below *x* axis. Ct values were calculated from nine biological replications in each group.

To preliminarily evaluate the expression of the candidate reference genes, the transcript abundances of these genes were estimated in normal, ARMs, and total samples. Only single peaks were found in RT-qPCR melting curves indicating that the specificity of the primers was good (Fig. 1). Based on the Ct values obtained from RT-qPCR, the expression profiles of the candidate genes in different samples were presented as the mean ± SD of the Cts in Fig. 2. Generally, the mean Ct values ranged from 14 to 22, mainly 14–17. Among all, Rplp1 was most abundantly expressed with lowest mean Ct value (14.07 ± 0.36), followed by Rpl13a (14.40 ± 0.41), Rps16 (14.87 ± 0.65), Rps18 (14.89 ± 0.29), Ppia (15.10 ± 0.53), Actb (15.10 ± 0.56), Gapdh (16.06 ± 0.44), Ubc (16.57 ± 0.25), Ywhaz (17.29 ± 0.43), Pgk1 (19.01 ± 0.63), B2m (19.20 ± 0.67), Hprt1 (21.36 ± 0.39), Hmbs (21.38 ± 0.40), Tbp (21.64 ± 0.62), and Sdha (21.89 ± 0.48). However, the stability of the candidate reference genes needs to be further explored.

### Stability analysis based on geNorm

**Figure 3:**
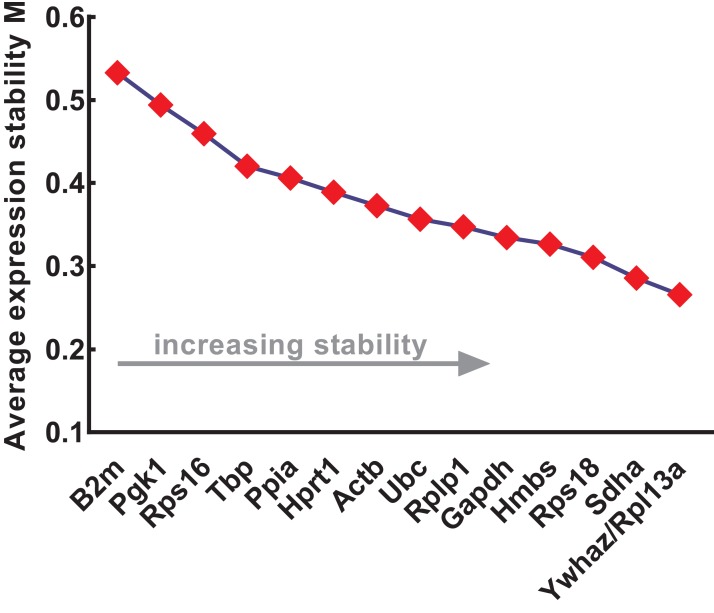
Stability of 15 candidate reference genes analyzed by geNorm. From left to right, reference genes are ranked according to increasing stability values (*M* values), ending with most stable gene pairs. N, normal; A, anorectal malformations. Input Ct values were calculated from nine biological replications in each group.

The expression stability of the candidate reference genes was further evaluated using four different methods: geNorm, NormFinder, comparative ΔCt, and BestKeeper. geNorm was used to calculate the stability values (*M* values) of each gene based on logarithmically transformed expression ratios and stepwise exclusion of the most unstable genes. As the *M* value decreased, the gene expression stability increased with improved ranking. Thus, the gene pairs with the lowest *M* values in the rank were most stably expressed. As shown in Fig. 3, B2m (*M* value = 0.533), Pgk1 (*M* value = 0.494), and Rps16 (*M* value = 0.459) were the most unstable genes, while Rpl13a/Ywhaz (*M* value = 0.266), Sdha (*M* value = 0.286), and Rps18 (*M* value = 0.311) were the most stable ones. Among these, Ywhaz/pl13a was the most stable gene pair.

### Stability analysis based on NormFinder

**Figure 4:**
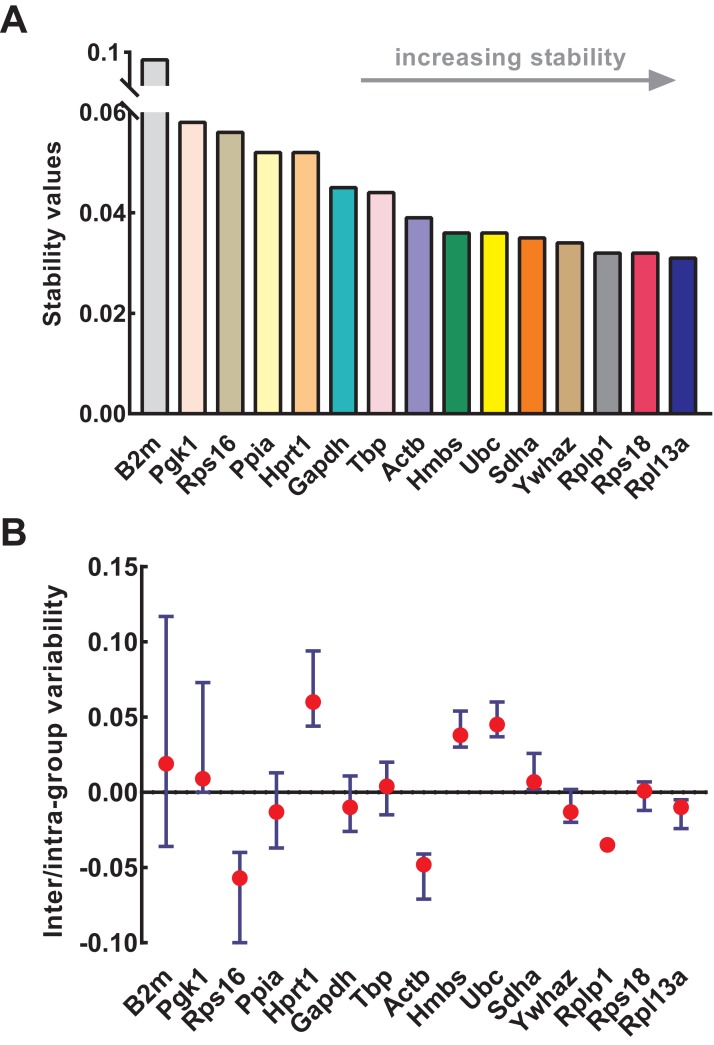
Stability of 15 candidate reference genes analyzed by NormFinder. (A) Expression stability values of reference genes. From left to right, reference genes are ranked according to increasing stability, ending with most stable gene pairs. (B) The intra- and inter-group variability. Error bars and red dots represent the intra- and inter-group variability, respectively. Input Ct values were calculated from nine biological replications in each group.

NormFinder was used to calculate the stability of genes as well as intra- and inter-group variations. Similar to geNorm, the input data of NormFinder was also logarithmically transformed. The gene stability values calculated by NormFinder are presented in Fig. 4A. As the stability value decreased, the gene expression stability increased with high ranking order. Similar to geNorm, B2m (stability value = 0.091), Pgk1 (stability value = 0.058), and Rps16 (stability value = 0.056) were the most unstable genes in NormFinder, further confirming their respective unstable expression. The four most stable genes were Rpl13a (stability value = 0.031), Rps18 (stability value = 0.032), Rplp1 (stability value = 0.032), and Ywhaz (stability value = 0.034). Slight differences occurred between geNorm and NormFinder. For example, Ywhaz ranked first in geNorm but fourth in NormFinder; and Sdha was included in four most stable genes in geNorm but not in NormFinder. Because of this discrepancy, various methods were necessary for the conjoint analysis while determining the optimal reference genes. The intra- and inter-group variations of each gene were also provided by NormFinder. As shown in Fig. 4B, Rps18 and Sdha also showed good stability with low inter- and intra-group variations of which variability values were close to zero.

### Stability analysis based on comparative ΔCt

The comparative ΔCt method gives ranking based on the relative pair, and the average SD was used to evaluate the expression stability of the genes. The larger the average SD, the lower was the stability ranking. As shown in Table 3, Rpl13a (0.423) was top-ranked, followed by Ywhaz (0.437), Rps18 (0.439), Hmbs (0.454), Sdha (0.458), Ubc (0.461), Rplp1 (0.471), Gapdh (0.490), Actb (0.517), and Hprt1 (0.527). In contrast, B2m (0.785), Pgk1 (0.726), Rps16 (0.703), Tbp (0.556), and Ppia (0.542) genes were ranked the last.

**Table 3:** Pairwise comparison for 15 candidate reference genes using comparitive ΔCt method.

Gene		RPS18	ACTB	B2M	GAPDH	PPIA	HPRT1	PGK1	YWHAZ	TBP	UBC	RPS16	RPL13A	RPLP1	SDHA	HMBS	Avg SD	Rank
Rpl13a	Mean	0.49	0.70	4.80	1.66	0.70	6.96	4.60	2.89	7.24	2.17	0.47	–	−0.33	7.49	6.98		
	SD	0.30	0.35	0.78	0.30	0.46	0.47	0.59	0.27	0.42	0.38	0.68	–	0.32	0.31	0.29	**0.42**	**1**
Ywhaz	Mean	−2.40	−2.18	1.91	−1.23	−2.19	4.07	1.72	–	4.35	−0.72	−2.41	−2.89	−3.22	4.60	4.09		
	SD	0.33	0.27	0.76	0.44	0.49	0.46	0.62	–	0.37	0.42	0.74	0.27	0.27	0.28	0.43	**0.44**	**2**
Rps18	Mean	–	0.22	4.31	1.17	0.22	6.47	4.12	2.40	6.75	1.68	−0.01	−0.49	−0.81	7.00	6.49		
	SD	–	0.41	0.68	0.40	0.48	0.39	0.67	0.33	0.51	0.30	0.66	0.30	0.30	0.38	0.33	**0.44**	**3**
Hmbs	Mean	−6.49	−6.27	−2.18	−5.32	−6.27	−0.02	−2.37	−4.09	0.26	−4.81	−6.50	−6.98	−7.30	0.51	–		
	SD	0.33	0.54	0.82	0.27	0.38	0.50	0.67	0.43	0.46	0.33	0.55	0.29	0.45	0.35	–	**0.45**	**4**
Sdha	Mean	−7.00	−6.79	−2.69	−5.83	−6.79	−0.53	−2.89	−4.60	−0.25	−5.32	−7.02	−7.49	−7.82	–	−0.51		
	SD	0.38	0.38	0.83	0.33	0.40	0.49	0.69	0.28	0.43	0.43	0.66	0.31	0.45	–	0.35	**0.46**	**5**
Ubc	Mean	−1.68	−1.47	2.63	−0.51	−1.47	4.79	2.44	0.72	5.07	–	−1.70	−2.17	−2.50	5.32	4.81		
	SD	0.30	0.53	0.56	0.43	0.51	0.28	0.71	0.42	0.59	–	0.59	0.38	0.40	0.43	0.33	**0.46**	**6**
Rplp1	Mean	0.81	1.03	5.13	1.99	1.03	7.29	4.93	3.22	7.57	2.50	0.80	0.33	–	7.82	7.30		
	SD	0.30	0.40	0.72	0.49	0.48	0.46	0.64	0.27	0.49	0.40	0.72	0.32	–	0.45	0.45	**0.47**	**7**
Gapdh	Mean	−1.17	−0.95	3.14	–	−0.95	5.30	2.95	1.23	5.58	0.51	−1.18	−1.66	−1.99	5.83	5.32		
	SD	0.40	0.54	0.92	–	0.37	0.59	0.64	0.44	0.53	0.43	0.60	0.30	0.49	0.33	0.27	**0.49**	**8**
Actb	Mean	−0.22	–	4.09	0.95	0.00	6.26	3.90	2.18	6.54	1.47	−0.23	−0.70	−1.03	6.79	6.27		
	SD	0.41	–	0.72	0.54	0.63	0.47	0.74	0.27	0.43	0.53	0.82	0.35	0.40	0.38	0.54	**0.52**	**9**
Hprt1	Mean	−6.47	−6.26	−2.16	−5.30	−6.26	–	−2.35	−4.07	0.28	−4.79	−6.49	−6.96	−7.29	0.53	0.02		
	SD	0.39	0.47	0.44	0.59	0.63	–	0.83	0.46	0.64	0.28	0.75	0.47	0.46	0.49	0.50	**0.53**	**10**
Ppia	Mean	−0.22	0.00	4.10	0.95	–	6.26	3.90	2.19	6.54	1.47	−0.23	−0.70	−1.03	6.79	6.27		
	SD	0.48	0.63	0.93	0.37	–	0.63	0.74	0.49	0.56	0.51	0.52	0.46	0.48	0.40	0.38	**0.54**	**11**
Tbp	Mean	−6.75	−6.54	−2.44	−5.58	−6.54	−0.28	−2.64	−4.35	–	−5.07	−6.77	−7.24	−7.57	0.25	−0.26		
	SD	0.51	0.43	0.93	0.53	0.56	0.64	0.65	0.37	–	0.59	0.79	0.42	0.49	0.43	0.46	**0.56**	**12**
Rps16	Mean	0.01	0.23	4.32	1.18	0.23	6.49	4.13	2.41	6.77	1.70	–	−0.47	−0.80	7.02	6.50		
	SD	0.66	0.82	0.85	0.60	0.52	0.75	0.91	0.74	0.79	0.59	–	0.68	0.72	0.66	0.55	**0.70**	**13**
Pgk1	Mean	−4.12	−3.90	0.19	−2.95	−3.90	2.35	–	−1.72	2.64	−2.44	−4.13	−4.60	−4.93	2.89	2.37		
	SD	0.67	0.74	1.06	0.64	0.74	0.83	–	0.62	0.65	0.71	0.91	0.59	0.64	0.69	0.67	**0.73**	**14**
B2m	Mean	−4.31	−4.09	–	−3.14	−4.10	2.16	−0.19	−1.91	2.44	−2.63	−4.32	−4.80	−5.13	2.69	2.18		
	SD	0.68	0.72	–	0.92	0.93	0.44	1.06	0.76	0.93	0.56	0.85	0.78	0.72	0.83	0.82	**0.79**	**15**

**Note:**

Avg SD, average standard deviation (SD) based on ΔCt of gene pairs for each reference gene; Ct, cycle threshold value; Rank, the stability of genes are ranked by Avg SD, and the higher the value, the lower the ranking.

### Stability analysis based on BestKeeper

BestKeeper determines the expression stability based on the pairwise correlation of all the candidate genes. Due to input limitation of BestKeeper, we ranked the candidate genes and selected the 10 most stable according to ranking orders of previous three methods (geNorm, NormFinder, and comparative ΔCt). Seven genes showed strong correlation, including Rpl13a (*r* = 0.921), Actb (*r* = 0.892), Ywhaz (*r* = 0.891), Sdha (*r* = 0.876), Rps18 (*r* = 0.786), Hmbs (*r* = 0.726), Rplp1 (*r* = 0.707), and Gapdh (*r* = 0.696) (Table 4). The other genes that showed moderate correlation were Ubc (*r* = 0.55) and Hprt1 (*r* = 0.51).

**Table 4:** Descriptive statistics for 10 candidate reference genes obtained by BestKeeper analysis.

Genes	*N*	GM (Ct)	AM (Ct)	Min (Ct)	Max (Ct)	SD (±Ct)	CV (% Ct)	*r* with BKI	*p*-value	Ranking order
Rpl13a	18	14.395	14.400	13.738	15.057	0.333	2.310	0.921	0.001	1
Actb	18	15.094	15.104	14.029	16.152	0.447	2.961	0.892	0.001	2
Ywhaz	18	17.283	17.288	16.617	18.134	0.349	2.017	0.891	0.001	3
Sdha	18	21.886	21.890	21.087	23.017	0.360	1.645	0.876	0.001	4
Rps18	18	14.883	14.886	14.236	15.355	0.214	1.438	0.786	0.001	5
Hmbs	18	21.373	21.376	20.691	21.999	0.327	1.530	0.726	0.001	6
Rplp1	18	14.068	14.072	13.609	14.948	0.278	1.973	0.707	0.001	7
Gapdh	18	16.052	16.057	15.320	16.950	0.340	2.118	0.696	0.001	8
Ubc	18	16.568	16.569	16.075	16.898	0.192	1.158	0.550	0.018	9
Hprt1	18	21.357	21.360	20.740	22.278	0.320	1.500	0.510	0.031	10

**Note:**

N, sample numbers; Ct, cycle threshold value; GM, geometric; AM, arithmetic; SD, standard deviation; CV, coefficient of variation; r, Pearson correlation coefficiency; BKI, BestKeeper Index; *p*-value, *p*-values associated with Pearson correlation.

### Comprehensive stability analysis of the candidate reference genes

**Figure 5:**
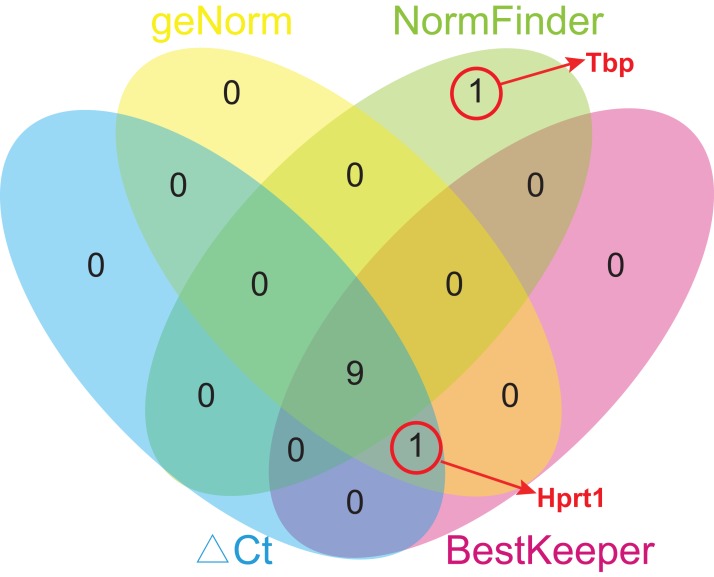
Venn diagram of top 10 stable genes obtained from geNorm, NormFinder, comparative ΔCt, and BestKeeper. Numbers in ellipses represent overlapped gene numbers. Tbp replaced Hprt1 in the top 10 gene list of NormFinder.

Due to different algorithms, different methods used in this study might have led to different ranking order. To avoid bias resulting from a single method and to obtain a more reliable result of gene stability, the comprehensive ranking orders based on four analyses were calculated using geometric means of the corresponding rankings of the top 10 genes. To avoid bias, only the genes common for at least three methods were chosen for further analysis, and Tbp was excluded from the comprehensive ranking because it appeared only in the top 10 gene list of NormFinder (Fig. 5). Rpl13a ranked first in every analysis, and so its comprehensive ranking order is still the highest, followed by Ywhaz (2.50), Rps18 (3.50), Sdha (4.25), Hmbs (5.50), Rplp1 (6.00), Actb (7.00), Ubc (7.25), Hprt1 (7.50), and Gapdh (8.00) (Table 5).

**Table 5:** Comprehensive ranking order of top 10 reference genes obtained from 4 different methods used in this study.

Ranking	ΔCt	geNorm	NormFinder	BestKeeper	Comprehensive ranking (mean rank value)
1	Rpl13a	Ywhaz/Rpl13a	Rpl13a	Rpl13a	Rpl13a (1.00)
2	Ywhaz		Rps18	Actb	Ywhaz (2.50)
3	Rps18	Sdha	Rplp1	Ywhaz	Rps18 (3.50)
4	Hmbs	Rps18	Ywhaz	Sdha	Sdha (4.25)
5	Sdha	Hmbs	Sdha	Rps18	Hmbs (5.50)
6	Ubc	Gapdh	Ubc	Hmbs	Rplp1 (6.00)
7	Rplp1	Rplp1	Hmbs	Rplp1	Actb (7.00)
8	Gapdh	Ubc	Actb	Gapdh	Ubc (7.25)
9	Actb	Actb	Tbp*	Ubc	Hprt1 (7.50)
10	Hprt1	Hprt1	Gapdh	Hprt1	Gapdh (8.00)

**Note:**

* Tbp was excluded from comprehensive ranking calculation because it appears only once in four analyses.

## Discussion

Real-time polymerase chain reaction is one of the most commonly used methods in gene expression analysis. It provides a simultaneous estimation of various gene expressions in different samples. However, many factors can affect the results of PCR, including the selection of the reference genes (Ferguson et al., 2010; Guo et al., 2013). An ideal reference gene should be stably expressed in all the samples without being affected by species, tissues, and development (Harris, Reeves & Phillips, 2009; Mughal et al., 2018). In fact, researches have revealed that no reference genes can be universally used under all conditions (Thellin et al., 1999). Therefore, analyses of the candidate reference genes are essential for accurate results in PCR. In this study, we analyzed 15 candidate RNA reference genes in the hindgut of normal and ARMs in rat fetuses aiming to determine the optimal reference gene for RT-qPCR.

In previous ARM-related studies, Gapdh and Actb were most commonly used as reference genes in RT-qPCR (Geng et al., 2017; Tang et al., 2018; Xiao et al., 2018). However, this study revealed that these two genes could be replaced by better candidates. In fact, six candidate reference genes were ranked before Gapdh and Actb in the expression stability including Rpl13a, Ywhaz, Rps18, Sdha, Hmbs, and Rplp1. Therefore, reference genes should be systematically evaluated, and only those that are relatively stable should be selected for further experiments.

While analyzing the gene expression stability, four different methods were mostly used, namely, geNorm, NormFinder, comparative ΔCt, and BestKeeper (Andersen, Jensen & Orntoft, 2004; Pfaffl et al., 2004; Silver et al., 2006; Vandesompele et al., 2002). In this study, nine (Actb, Gapdh, Rplp1, Ubc, Sdha, Hmbs, Rps18, Ywhaz, and Rpl13a) out of the top 10 stable candidate reference genes obtained by four methods overlapped. Out of the two remaining genes, Hprt1 appeared in of geNorm, BestKeeper, and Comparable ΔCt, while Tbp replaced Hprt1 in the top 10 list of NormFinder. However, discrepancies in the ranking orders by the four methods might have occurred because of the different computational principles followed. Thus, a comprehensive analysis of the multiple methods was further performed to reduce errors in screening optimal internal reference genes by a single method.

This comprehensive analysis was broadly used in various studies to identify the optimal reference genes (He et al., 2013; Klenke et al., 2016; Taki, Abdel-Rahman & Zhang, 2014). A comprehensive ranking order was generated by sorting the arithmetic mean of each gene’s rank order obtained from the four methods. The smaller the mean, the higher was the comprehensive ranking. A comprehensive analysis revealed that Rpl13a was the most stable reference gene followed by Ywhaz and Rps18. The commonly used Actb and Gapdh gene ranked seven and 10, respectively. These results indicated that Rpl13a was probably a better candidate for RT-qPCR normalization.

To the best of our knowledge, this is the first study to identify the optimal reference genes present in the hindgut of normal rat fetuses and those with ARMs. The results suggest the importance of systematically evaluating the expression stability of the candidate reference genes. However, there are some limitations to the present study. For example, RT-qPCR performed in this study was based on SYBR Green, and analyses between different experimental methods were not analyzed. The above problems are expected to be resolved in future studies.

## Conclusions

In conclusion, we identified 15 candidate RNA reference genes in the hindgut of normal and ETU-induced ARMs in rat fetuses and found that Rpl13a, Ywhaz, and Rps18 were most stably expressed genes. This result provides valuable information for future gene expression studies in ETU-induced ARMs.

## Supplemental Information

10.7717/peerj.6829/supp-1 Supplemental Information 1 Anatomic part of the samples used for RT-qPCR. The removed specimens are shown in the red dotted box. N, normal; A, anorectal malformations; U, urethra; R, rectum.

10.7717/peerj.6829/supp-2 Supplemental Information 2 Raw data of RT-qPCR used for analyses. N, normal group; A, ARMs group. RT-qPCR was performed for nine biological replications in each group.

## Additional Information and Declarations

### Data Availability

The following information was supplied regarding data availability: The raw measurements are available in Table S1.
