# Supplementary figures and images for: Identification of optimal endogenous reference RNAs for RT-qPCR normalization in hindgut of rat models with anorectal malformations
Source: PeerJ. 2019 Apr 23;7:e6829. doi: 10.7717/peerj.6829 (PMC6485207; doi:10.7717/peerj.6829)

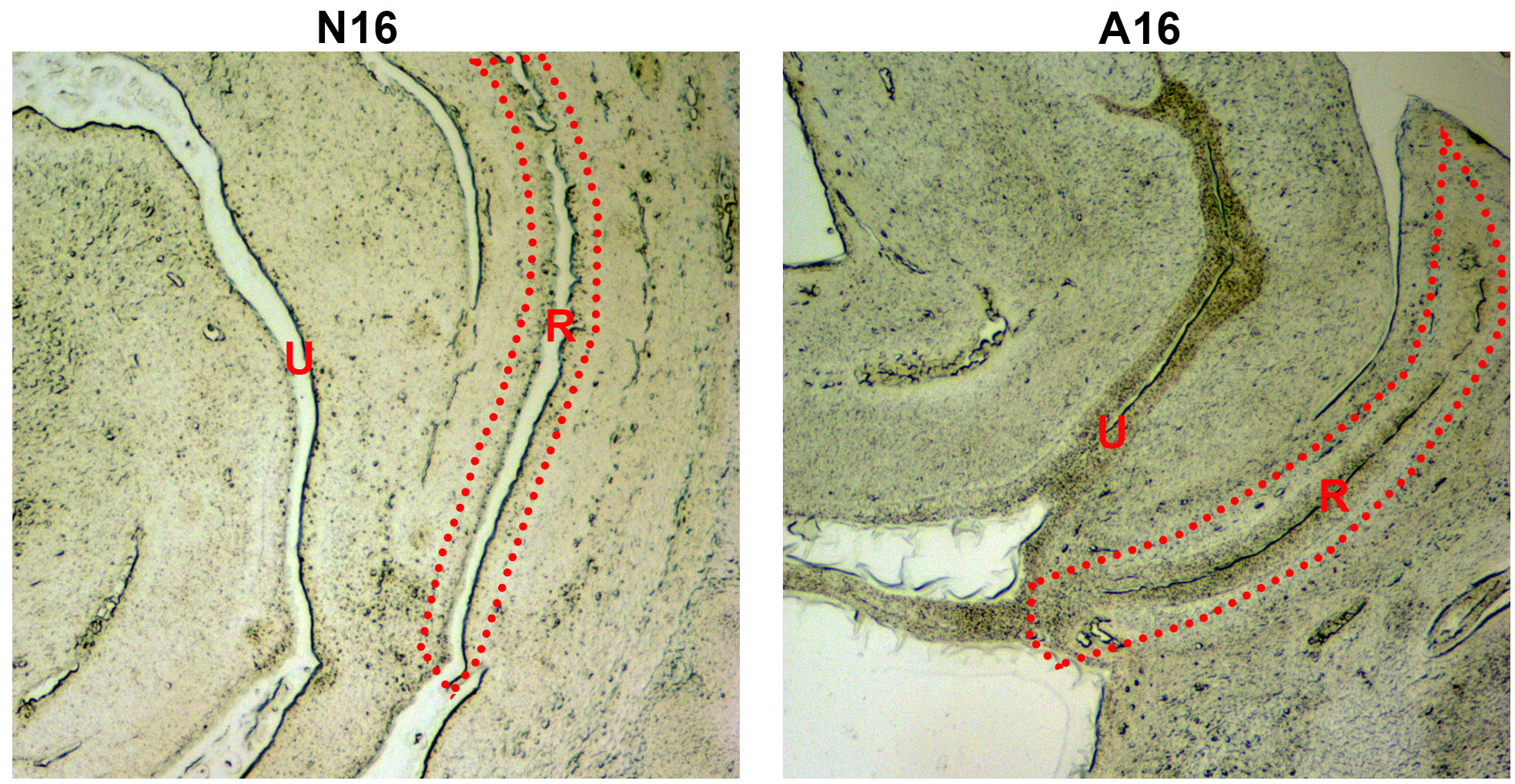

Supplement: Supplemental Information 1 — The removed specimens are shown in the red dotted box. N, normal; A, anorectal malformations; U, urethra; R, rectum. [file peerj-07-6829-s001.jpg]
