# Supplementary material for: Identification of optimal endogenous reference RNAs for RT-qPCR normalization in hindgut of rat models with anorectal malformations
Source: PeerJ. 2019 Apr 23;7:e6829. doi: 10.7717/peerj.6829 (PMC6485207; doi:10.7717/peerj.6829)
Supplement: Supplemental Information 2 — N, normal group; A, ARMs group. RT-qPCR was performed for nine biological replications in each group. [file peerj-07-6829-s002.docx]

|  | Rps18 | Actb | B2m | Gapdh | Ppia | Hprt1 | Pgk1 | Ywhaz | Tbp | Ubc | Rps16 | Rpl13a | Rplp1 | Sdha | Hmbs |
| --- | --- | --- | --- | --- | --- | --- | --- | --- | --- | --- | --- | --- | --- | --- | --- |
| A161 | 15.27937 | 15.4681 | 20.34224 | 15.98578 | 15.44179 | 21.74364 | 18.63653 | 17.4368 | 21.51522 | 16.79384 | 15.8545 | 14.49462 | 14.2267 | 22.20028 | 21.6304 |
| A162 | 14.8968 | 15.01296 | 20.06339 | 15.67501 | 14.85725 | 21.49778 | 18.08807 | 16.80274 | 20.91665 | 16.54498 | 15.10265 | 13.98965 | 14.04328 | 21.08735 | 20.94622 |
| A163 | 14.47865 | 15.52691 | 20.00828 | 15.45686 | 14.25715 | 21.70213 | 18.68873 | 17.59612 | 21.44258 | 16.60557 | 14.30634 | 14.26602 | 14.15215 | 21.76017 | 20.69114 |
| A164 | 14.65342 | 14.78771 | 18.84446 | 15.88078 | 14.80238 | 21.01525 | 18.43046 | 16.82387 | 21.15599 | 16.27538 | 15.26794 | 13.99338 | 13.64162 | 21.82116 | 21.22226 |
| A165 | 14.61417 | 14.92538 | 18.84898 | 15.97916 | 14.71537 | 20.73952 | 20.91346 | 17.14968 | 21.61614 | 16.30963 | 14.85263 | 14.32635 | 13.93799 | 21.52973 | 21.08785 |
| A166 | 15.24262 | 15.47036 | 18.78657 | 16.10045 | 15.13064 | 21.10312 | 18.90403 | 17.65432 | 21.72935 | 16.46803 | 14.30696 | 14.75001 | 14.94791 | 21.77431 | 21.30787 |
| A167 | 14.95289 | 15.17992 | 18.70225 | 16.94985 | 15.54433 | 21.27411 | 18.81049 | 17.28635 | 21.63723 | 16.64345 | 15.52239 | 14.42227 | 13.7597 | 22.44179 | 21.55992 |
| A168 | 14.9158 | 14.86625 | 18.70974 | 16.53346 | 15.81152 | 21.02504 | 19.38332 | 17.17244 | 21.63708 | 16.63089 | 15.88749 | 14.87312 | 14.13824 | 21.96116 | 21.82404 |
| A169 | 14.99659 | 15.51492 | 18.32761 | 16.26466 | 15.81608 | 21.03567 | 19.839 | 17.93423 | 22.73952 | 16.12226 | 14.84733 | 14.58853 | 14.63391 | 22.26917 | 21.38481 |
| N161 | 15.10232 | 15.46803 | 19.8745 | 15.97508 | 15.05289 | 21.80863 | 18.90765 | 17.416 | 22.89417 | 16.89751 | 15.33553 | 14.51882 | 14.18644 | 21.93289 | 21.90653 |
| N162 | 14.23628 | 14.02866 | 19.6819 | 15.5485 | 15.1154 | 21.54784 | 18.44086 | 16.61734 | 20.6941 | 16.62018 | 14.89063 | 13.73806 | 13.81503 | 21.23232 | 20.93313 |
| N163 | 15.35493 | 16.15181 | 20.30883 | 16.2539 | 14.94462 | 22.27755 | 19.4301 | 17.82802 | 22.14971 | 16.89372 | 14.3105 | 15.05665 | 14.29336 | 22.49102 | 21.51985 |
| N164 | 14.86896 | 14.77724 | 18.96451 | 15.95832 | 14.94224 | 21.294 | 19.02691 | 17.08701 | 21.53048 | 16.69503 | 14.43743 | 14.16986 | 13.94767 | 21.83734 | 21.42475 |
| N165 | 14.91662 | 14.3569 | 19.1827 | 15.31978 | 14.64534 | 21.02923 | 18.63195 | 17.06363 | 21.00579 | 16.4651 | 14.61195 | 13.76591 | 13.72119 | 21.53939 | 20.90612 |
| N166 | 14.71149 | 14.76263 | 18.49623 | 15.73218 | 14.17478 | 21.14356 | 18.74392 | 16.78576 | 21.02572 | 16.07457 | 13.25142 | 14.17913 | 13.60933 | 21.37058 | 20.97228 |
| N167 | 14.72048 | 14.46865 | 18.34595 | 16.09231 | 15.18017 | 21.28046 | 19.03844 | 16.95206 | 21.31377 | 16.606 | 14.36541 | 14.17895 | 13.7853 | 21.71102 | 21.53668 |
| N168 | 14.87817 | 15.02845 | 18.9304 | 16.66506 | 15.1441 | 21.13555 | 19.06593 | 17.44054 | 21.96544 | 16.77251 | 15.35204 | 14.87365 | 13.96046 | 22.05088 | 21.99913 |
| N169 | 15.12875 | 16.07818 | 19.13996 | 16.65989 | 16.26992 | 21.82622 | 19.11311 | 18.13438 | 22.56423 | 16.83141 | 15.20996 | 15.01801 | 14.49729 | 23.01698 | 21.92157 |
